# Research on the Application of MWCNTs/PLA Composite Material in the Manufacturing of Conductive Composite Products in 3D Printing

**DOI:** 10.3390/mi9120635

**Published:** 2018-11-30

**Authors:** Jinjie Luo, Haibao Wang, Duquan Zuo, Anping Ji, Yaowen Liu

**Affiliations:** 1 Department of Mechanical Engineeering, Chongqing Sanxia Institute, Chongqing 404000, China; luoke1949@126.com (J.L.); Wanghbw@21cn.com (H.W.); taiyanghefeng@126.com (D.Z.); japmail721@163.com (A.J.); 2 Chongqing Engineering Technology Research Center for Light Alloy and Processing, Chongqing 404000, China; 3 School of Materials Science and Engineering, Southwest Jiaotong University, Chengdu 610031, China; 4 College of Food Science, Sichuan Agricultural University, Yaan 625014, China

**Keywords:** PLA, MWCNTs, 3D printer

## Abstract

As an advanced manufacturing technology that has been developed in recent years, three-dimensional (3D) printing of macromolecular materials can create complex-shaped components that cannot be realized by traditional processing. However, only a few types of macromolecular materials are suitable for 3D printing: the structure must have a single function, and manufacturing macromolecular functional devices is difficult. In this study, using poly lactic acid (PLA) as a matrix, conductive composites were prepared by adding various contents of multi-walled carbon nanotubes (MWCNTs). The printability and properties of MWCNT/PLA composites with different MWCNT proportions were studied by using the fused deposition modeling (FDM) processing technology of 3D printing. The experimental results showed that high conductivity can be realized in 3D-printed products with a composite material containing 5% MWCNTs; its conductivity was 0.4 ± 0.2 S/cm, its tensile strength was 78.4 ± 12.4 MPa, and its elongation at break was 94.4% ± 14.3%. It had a good melt flow rate and thermal properties, and it enabled smooth printing, thus meeting all the requirements for the 3D printing of consumables.

## 1. Introduction

Three-dimensional (3D) printing differs from traditional manufacturing processes. It enables the physical realization of a 3D computer-aided design (CAD) model via the layer-by-layer addition of materials, which results in a 3D physical object that has the same structure as the digital model. 3D printing has received widespread attention in many different industrial fields as a representative technique from the Fourth Industrial Revolution (Industry 4.0). Currently, 3D printing is widely used in biological tissue engineering, medicine, environment, and food applications [1]. Compared with traditional processing methods, it can greatly reduce the cost of processing products, shorten prototyping lead time, and avoid material waste. In addition, its manufacturing principles, such as the facile processing of complex surfaces, immediate material shaping, mold-free fabrication, and integrated design and manufacturing, result in an extremely high material utilization rate, as well as convenient and quick production [2]. However, the shortcomings of 3D printing, such as single consumption, low product strength, and limited application range, inhibit the wide application of 3D printing technology in various industries [3].

In the plastic industry, traditional single-material printing cannot meet the industrial requirements due to the increasing demands for complex and multi-functional plastic products. Nowadays, the application of macromolecular composite materials is increasingly widespread, and many special materials’ properties can be realized by combining organic and inorganic materials [4]. Conductive materials, antistatic materials, electromagnetic shielding materials, digitally controlled heating materials, and other conductive composite materials have attracted the attention of researchers worldwide. Conductive macromolecular composite materials are prepared by incorporating conductive fillers (such as carbon nanotubes, carbon fibers, etc.) into a single-phase or multiphase macromolecular system [5]. Haggenmueller et al. obtained a carbon nanotube/polymethyl formamide composite membrane by melt blending. The addition of carbon nanotubes significantly improves the electrical and mechanical properties of materials [6]. Kumar produced a carbon nanotube/polypropylene fiber composite material through melt spinning and found that the addition of 5% carbon nanotubes into polypropylene increased the elastic modulus and mechanical strength of the composite by 50% and 100%, respectively [7]. However, because polymers can degrade at certain temperatures and materials need to be melt and blended several times to achieve uniform mixing, heat-resistant and thermally stable polymers are required for forming composites with multi-walled carbon nanotubes (MWCNTs) [8].

Some researchers have proposed a solution mixing method in which carbon nanotubes are dispersed in a suitable solvent and then the dispersion is mixed with a polymer [9]. Finally, the carbon nanotube/polymer composite is manufactured through casting, drying, and other processes at selected temperatures. This method has the advantage of ease of operation and fast material mixing speed; however, it has the disadvantage that during the solvent evaporation, the carbon nanotubes can easily coalesce, resulting in their uneven dispersion in the composite material [10]. In order to improve the dispersion of carbon nanotubes in materials, a surfactant is usually employed to modify the carbon nanotube surface to render the surface of the carbon nanotubes static, or hydrophilic groups (such as –OH) are formed on the surface [11]. Shaffer et al. used the chemical modification method for surface modification of carbon nanotubes. The modified carbon nanotubes have static electricity, and the carbon nanotubes could be uniformly dispersed in a polyvinyl alcohol matrix, thus yielding composite materials with stable performances [12]. Although the material can be formed at one time, it does not require high-temperature heat treatment, thus avoiding the thermal degradation of the polymer materials, and the dispersion degree of the carbon nanotubes is very high. However, its disadvantage is that the polymer molecular weight is not high. Moreover, the abovementioned techniques can only fabricate simple composites of poly lactic acid (PLA) and MWCNTs but fail to complete the later shaping phase.

There are few reports on carbon nanotubes and macromolecular materials for 3D printing, and more than 90% of the consumables currently in the market are acrylonitrile butadiene styrene (ABS) and PLA engineering plastics [13]. The 3D printing technology has realized the efficient preparation of high-performance conductive fibers and conductive fiber network structures. Li et al. proposed nanocellulose as a dispersant for carbon nanotubes in aqueous solutions and a reinforcing agent for conductive fibers to improve the performance of conductive fibers, ensure manufacturing safety, and reduce costs. Their results revealed that, as a dispersant, nanocellulose can successfully disperse carbon nanotubes in aqueous solutions and eliminate the use of organic solvents with better safety and lower cost. Further, the rheological property of carbon nanotube dispersions dispersed by nanocellulose can be adjusted. For example, shear thinning has been observed at 0.5% dispersion concentration. Moreover, as a reinforcing agent, nanocellulose improves the mechanical properties of conductive fibers [14]. Therefore, with the 3D printing technology it is possible to improve the conductivity of composite materials, ensure good mechanical and conductive properties, and facilitate large-scale and efficient production.

At present, the 3D printing technology mainly includes a stereolithography apparatus (SLA), fused deposition modeling (FDM), and selective laser sintering (SLS) among other features [15]. SLA is the most widely applied technique owing to certain attributes such as higher automation during forming, good printed prototype surface quality, high size accuracy, and realizable fine size forming [16]. However, its disadvantages include limitations of material availability and application surface. SLS is often used to fabricate structural functional parts, and the laser beam selectively sinters fused powder material such as nylon, elastomeric materials, and metallic materials [17]. FDM is used to extrude wire materials as thermoplastic plastics, wax, metallic fuse out of heated head, and for melt deposition at a fixed rate according to a predetermined track of two-dimensional (2D) data after a part section. The principle and operation of the FDM system are simple; thus, it is among the most common and mature 3D printing technology [18]. FDM printing consumables have strict requirements for wires. The material is coiled into the head through gears, and the distance between the gears and the fixed wheel is constant. If the wire is too thick, it will cause wire feeding failure or damage the wire feeding mechanism. On the contrary, if it is too fine, the wire feeding mechanism will not sense it.

In this paper, the FDM process of 3D printing technology is used to study a MWCNT/PLA composite material and its preparation method, which can be used for 3D printing. In addition, the effects of MWCNTs on the conductivity, thermal reanalysis, melt flow rate, and morphology of the produced composite material are studied, and a process for printing MWCNT/PLA composite materials with a 3D printer is proposed. Because of their outstanding electrical and mechanical properties, incorporating conductive particles into such composites opens the way to advanced conducting materials for energy storage, biosensors, and tissue engineering [19].

## 2. Materials and Methods

### 2.1. Materials

PLA was composited in the lab with a molecular weight of 200,000. MWCNTs (7–15 nm in diameter, 5–10 mm in length, and purity of >97%) were purchased from the Chengdu Institute of Organic Chemistry, Chinese Academy of Sciences. All other chemicals and solvents were of reagent grade or better and purchased from Chengdu Kelong Reagent Co. (Chengdu, China), unless otherwise indicated.

### 2.2. Preparation of the MWCNT/PLA Composite Material

In this experiment, the composition of MWCNTs of fillers and polymer poly lactic acid (PLA) were achieved by mechanical agitation. Composite materials of MWCNTs and PLA with different mass fractions of 1, 3, 5, 7, and 9% were prepared by mechanical agitation. First, the PLA aggregates were dried in a hot air circulation-drying oven at 50 °C to remove excess water. According to predetermined proportions, the components were accurately measured, and the PLA aggregates were dissolved in an appropriate amount of chloroform solvent with magnetic stirring for 10 h in a sealed environment. After complete dissolution, MWCNTs were added and continuously stirred for 4 h for standby application. This was the solution mixing process. The obtained mixture was placed into a glass dryer for 15–20 min and dried in a constant temperature drying oven at 90 °C to obtain a cake mixture. The cake mixture was then cut into square blocks with dimensions of 2 mm × 2 mm × 2 mm for melt blending. A self-assembled double-screw extruder was used to shear and extrude the mixed material with the temperature controlled between 150 °C and 185 °C at a screw speed of 80 r/min. Finally, a MWCNT/PLA composite wire with a diameter of 1.75 ± 0.05 mm was obtained after the cooling and traction of the melt.

### 2.3. 3D Printing of the MWCNT/PLA Composite Material

For the 3D printing, an FDM printer from Chengdu Pulian Co. (Chengdu, China), firstly, UG8.0 software (Siemens PLM Software, Plano, TX, USA) was used to draw 2D and 3D physical models of the sample, followed by slicing using the slice software (simplify 3D) [20]. The relevant printing parameters were set as follows: head diameter, 0.4 mm; thickness, 0.3 mm; filling rate, 100%; processing temperature, 210 °C; and printing rate, 9 mm/s. An A4 paper was tiled out and fixed on the printing platform, onto which the head dispensed the material, and the 3D model was formed layer by layer.

### 2.4. Composite Material Performance Test Method

Scanning electron microscopy (SEM, FEI Quanta 200, Amsterdam, The Netherlands) was used to visualize the structural topography of the composite materials, which were sputter-coated with a thin layer of gold to minimize the charging effect. The encapsulated MWCNTs within the PLA were observed via transmission electron microscopy (TEM, Hitachi H-700H, Tokyo, Japan) at an electron acceleration voltage of 75 kV [21]. The tensile strength and the elongation at break of the specimens were tested using a universal test machine (Instron 5567, Instron Co., Norwood, MA, USA) at a stretch speed of 2 mm/min and a load cell of 5 N. Next, five separate runs were performed on each sample, and the Young’s modulus and elongation at the break were obtained from the stress–strain curves. The Young’s moduli for the composite materials were obtained from the slope of the stress–strain curve in the linear region. The electrical conductivity of the composite was measured using a SX1934 digital four-probe tester (Suzhou Baishen Technology Co., Ltd., Suzhou, China), and the resistivity of the 3D-printed conductive composite was measured using the four-electrode method [22]. A composite filament with a diameter of 1.75 mm was used to form an electric current loop at both ends of the electrode, and the two electrodes were attached to the center of the filament to form a voltage test circuit. The resistivity of the material is calculated according to the following equation:

*ρ* = *R*A/*L* = *U*A/*IL*

where *ρ* is the volume resistivity of the sample (Ω·cm), *U* and *R* are the voltage (V) and resistance generated by the test current *I* (A) after passing a certain distance *L* (cm) of the sample, respectively, and *A* is the cross-sectional area (cm^2^) of the test sample. The volume conductivity of the sample (*κ*, S/cm) is calculated according to the following equation:

*κ* = 1/*ρ*

The melt flow rate (MFR) is the quality of the thermoplastic plastic at the specified temperature and load through the standard mouth mold every 10 min (g/10 min) [23]. It is often used as an important index to measure the fluidity of a plastic melt and was measured using a Melt Flow Testers (Instron^®^’s CEAST Melt Flow Tester, Instron, Norwood, MA, USA). For the same polymer under the same load and temperature, the larger the MFR, the lower the average molecular weight of the polymer and the better the fluidity of the plastic melt for the thermal forming process [24].

Thermogravimetric analysis is a kind of thermal analysis technique, which is used to measure the quality of samples with the change of temperature under the control of programs. Thermogravimetric analysis is used to study the thermal stability and components proportional composition of materials [25]. The thermal degradation of the films was monitored with a thermogravimetric analyzer (TGA, TGA-Q500, TA Instruments, New Castle, DE, USA). The sample was first dried to prevent the effects of moisture in the sample. Then, a mass of 3~5 mg of the sample was placed in the sample chamber and heated from room temperature to 500 °C at a rate of 10 °C min^−1^, and the TGA weight loss curve was recorded.

Vickers hardness tester was used to measure the hardness with a load setting of 5KN and dual time of 15 s [26]. A Zwick/Z010 tensile tester (Zwick Roell Group, Ulm, Germany) was used to test the adhesion according to the standard test method SS-EN ISO 11339:2010. The separation rate was 100 mm/min [27]. The hardness and adhesion were measured from 5 different samples, and the average value was taken.

### 2.5. Statistical Analysis

The statistical significance of the rheological data between the selected samples was assessed via independent t-test with a significance level of 0.05 by using SPSS Statistics 22 (SPSS Inc., Chicago, IL, USA) software.

## 3. Results and Discussion

### 3.1. Dispersion of Materials

**Figure 1:**
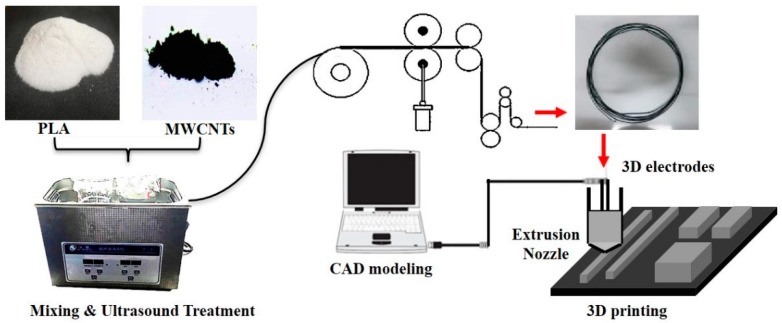
The fabrication process of poly lactic acid (PLA)/multi-walled carbon nanotubes (MWCNTS) composite materials by three-dimensional (3D) printing.

Carbon nanotubes used in the preparation of composite materials should have good dispersibility and compatibility. Because carbon nanotubes are one-dimensional nanomaterials with very small diameters and a high surface energy, they often aggregate and wrap around. This affects their dispersion in the polymer matrix, which inevitably leads to performance degradation. This study employed self-made equipment, as shown in Figure 1. First, the filament raw material was transported to the hot runner by a feeding roller. After the composite material entered the head smoothly, it was extruded and printed at the port. If the hardness of the composite material was insufficient, it would not have been able to provide enough pressure to smoothly feed the wire into the head [28]. Moreover, it has been found that it is difficult to prepare MWCNT/PLA samples with a mass ratio greater than 7 wt% using a 3D printer with this structure.

**Figure 2:**
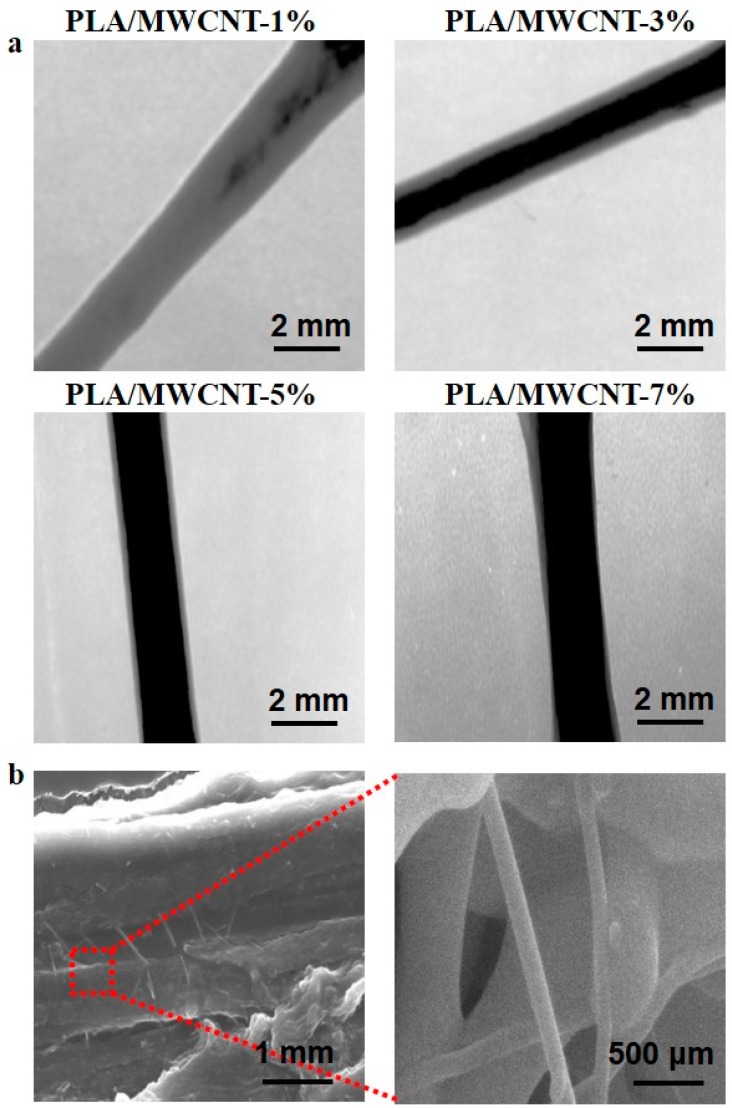
(**a**) Transmission electron microscopy (TEM) images of PLA containing different amounts of MWCNTs. (**b**) Typical scanning electron microscopy (SEM) images of the cross-sections of PLA containing 5% MWCNTs.

The dispersion of MWCNTs in the PLA matrix was observed using TEM, and the results are shown in Figure 2a. The MWCNTs with mass ratios of 1% were unevenly distributed in the PLA matrix, whereas those with mass ratios of 3% and 5 wt% in the samples were uniformly dispersed in the PLA matrix without agglomeration. It can be seen that the carbon nanotubes were interlocked with the matrix, and most of them were well dispersed in the matrix. This occurred because during the preparation of the composite material, the polymer molecular chains on the surface of the carbon nanotubes continuously increased, and finally, a PLA coating layer was formed on the surface of the carbon nanotubes [29]. When the MWCNT content was greater than 7 wt%, the MWCNTs extracted from the section were not adhered to the PLA matrix, indicating that the compatibility between the MWCNTs and the PLA matrix was not very good.

The section diagram of Figure 2b also shows that the carbon nanotube wall was evenly coated with PLA, and the MWCNTs and PLA had a very good interface and could not be easily separated. Further, most of the carbon nanotubes were dispersed uniformly in the PLA matrix, with the ends of the carbon nanotubes buried inside the matrix and some sections of the carbon nanotubes exposed. Moreover, although the MWCNTs in the rough section were pulled out in 5 wt% composite wires, there was no obvious separation between the MWCNTs and the PLA matrix. It can be predicted that because of the van der Waals force between the MWCNTs and PLA, the two may form a stable compatible structure [21].

### 3.2. Electrical Conductivity of the Materials

**Figure 3:**
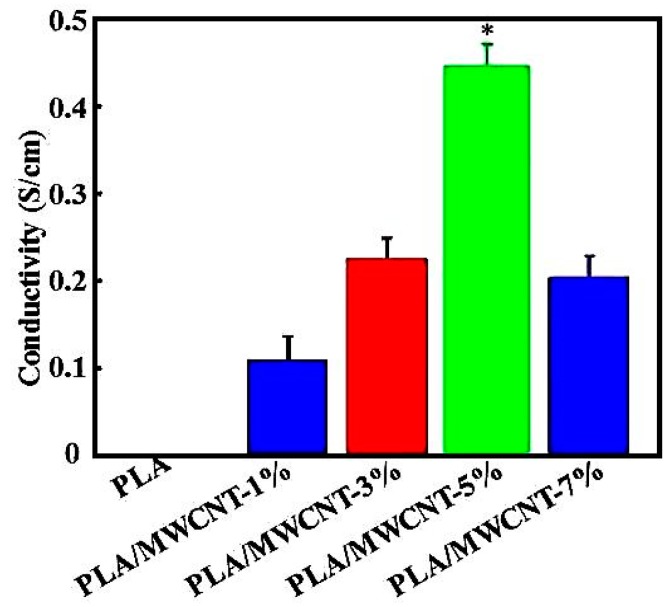
Conductivity of the PLA containing different amounts of MWCNTs.

Figure 3 shows the influence of different MWCNT contents on the conductivity of the composite materials. The electric conductivity of the pure PLA after the 3D printing was 1.06 × 10^−15^ S/cm, which classifies it as an insulation material, and the electric conductivities of the composite materials with 1%, 3%, 5%, and 7% MWCNT contents were (0.1 ± 0.1) S/cm, (0.2 ± 0.1) S/cm, (0.4 ± 0.2) S/cm, and (0.2 ± 0.1) S/cm, respectively. This shows that after the MWCNTs were rearranged in the PLA, the homogeneity and content of the MWCNTs were improved in the composites. In the PLA matrix, the MWCNTs formed a continuous conducting network, thus providing a relatively stable electrical conductivity, which met the performance requirements of a conductive body for various applications. At 7% MWCNT content, the conductivity of the composite material decreased but was still sufficiently good. For a relatively high MWCNT content, after the preparation of the composite material via 3D printing, the penetration rate of the electron was affected due to the uneven distribution of the MWCNTs and their poor adhesion with the PLA. Liu et al. have also prepared MWCNT/PLA composite materials and found that with the addition of carbon nanotubes the electromagnetic shielding efficiency of the composites increased. When the carbon content reached 7%, the conductivity was the highest [21]. Deng et al. prepared polypropylene (PP)/carbon nanotubes (CNT) composite materials with layered structures via the melting method and significantly improved the conductivity (up to 275 S/cm) while ensuring a high tensile strength of over 50 MPa [30]. Flowers et al. investigated the use of dual-material fused filament fabrication for 3D printing electronic components and circuits with conductive thermoplastic filaments. They found that the resistivity of traces printed from conductive thermoplastic filaments made with carbon-black, graphene, and copper as conductive fillers was 12, 0.78, and 0.014 Ω·cm, respectively [31]. These resistivities were on the same level as the resistivities of traces obtained in the present research.

Furthermore, we found that for the same proportion of MWCNT in the MWCNT/PLA composite, the resistivity of the extruded wire was significantly lower than that of the 3D printed products, because during the 3D printing process, after 0.4 mm head heating extrusion, the Φ1.75 mm wire changed the distribution of the MWCNTs in the PLA. In addition, because the melting point of the MWCNTs is extremely high, and the PLA melting point is relatively low [32], during heating in the 3D printing process, the PLA plasticization became flow dynamic, while the MWCNTs were still solid; thus, the distribution of the MWCNTs in the PLA changed, causing an increase in resistivity. Therefore, 3D printing with the FDM process has an important influence in improving the performance of the composite materials.

### 3.3. Mechanical Properties and Application of the MWCNT/PLA Composite Materials

Table 1 shows the tensile strength test results of different composite materials after extrusion and 3D printing. With the increase in the MWCNT content, the stress resistance of the composites gradually increased. For the pure PLA the tensile strenght increased from (18.8 ± 2.1) MPa to (78.4 ± 12.4) MPa (5% of MWCNTs), which indicates that the 5% of MWCNTs are not only dispersed uniformly in the PLA matrix, but also well combined with the PLA matrix to bear certain tensile stress loads. The study shows that after adding the wild phase, the tensile strength of the composite depended on whether the wild phase was uniformly distributed in the matrix resin to some extent, as well as on the interface morphology and bonding state [33]. Therefore, the tensile strength of the composites increased with the addition of MWCNTs into the PLA. However, when the content of the MWCNTs exceeded 7%, the mechanical properties of the MWCNTs deteriorated due to their poor dispersion; however, the performance of the polymer materials could be greatly improved. Qian et al. also reported the preparation of carbon nanotube/polystyrene composites by solution evaporation. When the carbon nanotube content was 1 wt%, the elastic modulus of the composite film increased by 36–42%, and the impact strength increased by about 25% [34].

The variation in the elongation at the break was consistent with the result of the tensile strength analysis. The elongation at the break of the pure PLA was 26.2% ± 3.4%. When the MWCNT content was 5%, the elongation at the break of the composite material reached a maximum of 94.4% ± 14.3%. As the content of MWCNT increased, the Young’s modulus of the composite material increased. The increase in the MWCNT contents from none to 5% led to significant increases in Young’s modulus from (78.4 ± 10.3) MPa to (134.4 ± 24.3) MPa for PLA/MWCNT-5%. When the amount of MWCNT increased to 7%, the continuity of the whole matrix changed, because the increase in the MWCNT was more likely to cause agglomeration, which was not conducive to deformation, energy transfer, or diffusion of the matrix under stress [35], nor could it stop cracking or generate cracks to absorb the impact energy. On the contrary, it mainly caused stress concentration in the matrix, thus decreasing the toughness and increasing the brittleness of the composites. Carson et al. also reported that carbon nanotubes could enhance the mechanical properties and toughen the nanotubes owing to their large size and small density [36].

### 3.4. Analysis of Melt Flow Rate and Thermal Performance (MFR) of the MWCNT/PLA Composite Materials

If the MFR is too low, the extrusion of the wire from the head for printing becomes difficult [37]. On the other hand, if the MFR is too high, material will leak out and affect the printing quality. As can be seen from Table 2, the MFRs of the composite materials do not show a specific trend. The MFR of the pure PLA wire was 18.8 g/10 min, and it increased after the incorporation of the carbon nanotubes (no significant difference) in the PLA matrix. When the content of the carbon nanotubes was relatively small (1% to 5%), the MFR of the material remained almost the same, thus indicating no significant change in the composite material before or after extrusion during 3D printing. It also reflected that when the concentration of MWCNTs was between 1% and 5%, the composite and pure PLA had similar melt flow rates, suitable for the processing and 3D printing of the composite materials. For the composite with 7% MWCNTs, because of the high carbon nanotube content, the MFR of the composite material decreased, and the wire extrusion from the head for printing became difficult [38].

**Figure 4:**
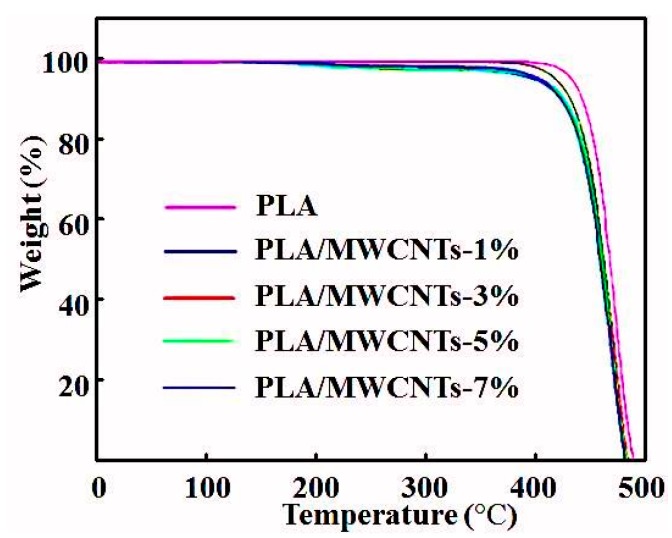
Thermogravimetric analysis (TGA) thermograms of PLA and the PLA/MWCNTs composite materials.

Figure 4 shows the TGA curves of the MWCNT/PLA composite wires with various MWCNT contents. The TGA curve of the pure PLA and MWCNT/PLA composite wires were quite similar. It can be seen that the addition of the MWCNTs reduced the thermal decomposition temperature of pure PLA. As the MWCNT content increased, the initial decomposition temperature remained almost unchanged. We speculated that the extremely low content of MWCNTs in the composite wire had an insignificant effect on the thermal decomposition temperature and thermal stability of the pure PLA, and thus, the TGA data were almost unchanged. The thermal decomposition temperature of the finished wire was the highest, because the chain-extended pure PLA was cross-linked, branched, and chain expanded [39], which increased the molecular weight and initial decomposition temperature, thereby improving the thermal stability.

### 3.5. Printing of Consumables and Printing Effect

**Figure 5:**
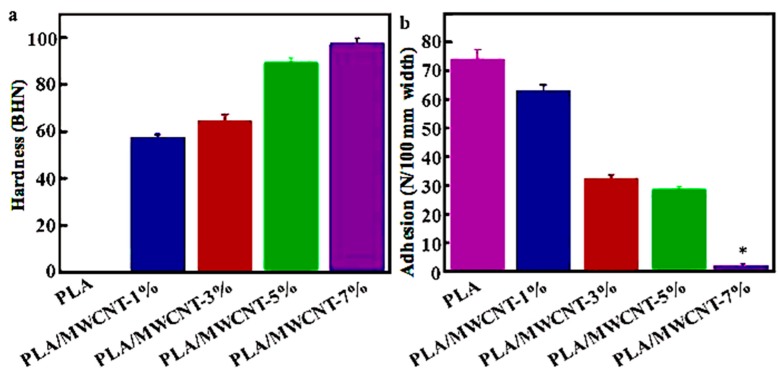
(**a**) Hardness, (**b**) adhesion of the extruded PLA/MWCNTs samples containing different amounts of MWCNTs.

As shown in the Figure 5a, adding the MWCNT can effectively increasing the hardness of the printed consumable of the MWCNT/PLA composite materials, because the addition of the MWCNT powder significantly increased the hardness of the MWCNTs/PLA composite materials. This was partially due to the reduced mass of the PLA and the higher content of the MWCNTs in the composite material, which promoted the binding of MWCNT together. Such behavior suppressed the PLA network. Consequently, the MWCNT/PLA composite material became stiff. The addition of the MWCNT substances simultaneously reduced the adhesive property of the MWCNT/PLA composite materials (Figure 5b). Adding PLA could improve the adhesion performance of the MWCNT/PLA composite materials. Mixing a certain amount of MWCNT could decrease the distribution uniformity of the PLA and form a MWCNT network and microstructure inside the MWCNT/PLA composite material product, which lowered the adhesion force; further, this would ultimately reduce the ability of the product to maintain its geometry.

**Figure 6:**
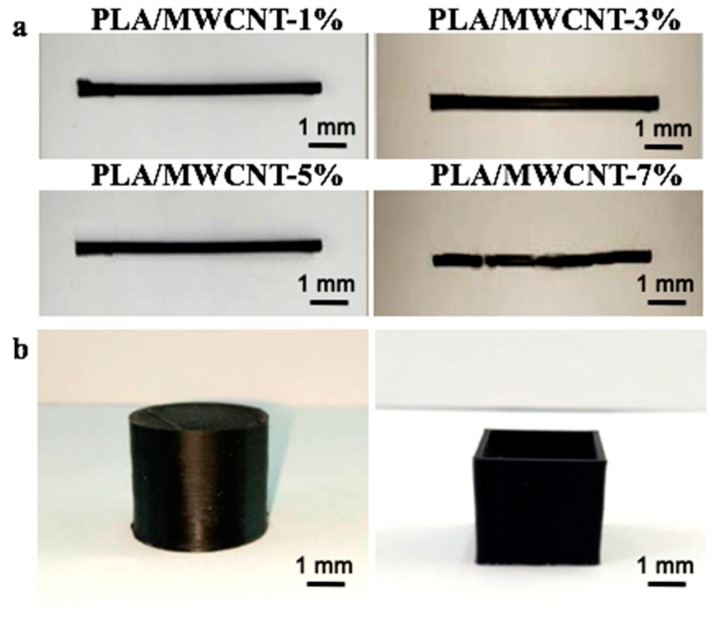
(**a**) Line test of the extruded PLA/MWCNTs samples containing different amounts of MWCNTs; (**b**) Pictures of some products printed with a 0.4-mm nozzle diameter.

Figure 6a shows the printed consumables of the MWCNTs/PLA composite materials prepared according to different proportions. In addition to the 7% contents of the broken wires in the preparation process of the consumables, 1%, 3%, and 5% contents could produce volume print wires without any breaking off or broken wire during production. This implies that the toughness of the composite materials met the requirement for 3D printing of the consumable materials. In addition, the surfaces of the conductive 3D-printed MWCNT/PLA composite consumables were much rougher than those of the pure PLA 3D-printed consumables, because the high content of the MWCNTs in the conductive 3D-printed consumables affected their surface quality, which was consistent with the scanning electron microscopy results. The line product of PLA/MWCNT-7% was degraded significantly (*p* < 0.05) after undergoing 3D printing. This degradation occurred because the 3D printing process first involved the extrusion of the material and then the execution of a specific sequence. Therefore, the products were not mixed together, but rather adhered together with a weaker interaction. In other words, the products were only in physical contact with each other instead of being merged together during printing. Consequently, broken wires occurred in the line product of PLA/MWCNT-7% during production, because the adhesive property of the PLA/MWCNT-7% was reduced significantly (*p* < 0.05) (Figure 5b).

Figure 6b shows the printing effect on the conductive finished products of the 3D-printed consumables with 5% MWCNT. The test spline and cube printed by the printer were not different from the test cylinder (left) and cube (right) printed using the general PLA printing consumables, which had good printing and shaping effects, smooth surface, smooth printing process, and no print-head blockage or edge warping. The relevant printing parameters were a 0.4-mm head diameter, 0.3-mm thickness, a 100% filling rate, a processing temperature of 210 °C, and a 9 mm/s printing rate, which met the printing requirements of most 3D printers for consumables.

## 4. Conclusions

By combining melt compounding with the FDM technology, MWCNT/PLA composite wires with different MWCNT contents were prepared. Their microstructure, electrical conductivity, mechanical properties, rheological property, and thermal properties were investigated. When the MWCNT content was less than 5 wt%, the surface of the composite wire was relatively smooth. MWCNTs as fillers significantly enhanced the conductivity and mechanical properties of the composites, and the flow rate and thermal performance of the composites were stable. The MWCNTs may have formed a stable compatible structure in the PLA matrix. Test samples obtained through 3D printing could be effectively produced in shapes such as bar, line, cylinder and cube-like geometries. In summary, the prepared MWCNT/PLA composite wire met the requirements of most 3D printers, which can significantly enable the manufacture of conductive composite products by 3D printers in the future.

## Figures and Tables

**Table 1:** Mechanical properties of PLA and the PVA/MWCNTs composite materials (*n* = 6).

Properties	PLA	PVA/MWCNT-1%	PVA/MWCNT-3%	PVA/MWCNT-5%	PVA/MWCNT-7%
Stress (MPa)	18.8 ± 2.1	44.7 ± 8.4	67.2 ± 10.3	78.4 ± 12.4	72.2 ± 11.4
Strain (%)	26.2 ± 3.4	78.6 ± 12.7	90.1 ± 13.3	94.4 ± 14.3	91.9 ± 13.8
Young’s Modulus	78.4 ± 10.3	108.5 ± 17.4	127.6 ± 21.0	134.4 ± 24.3	114.8 ± 18.2

**Table 2:** Melt flow rate (MFR) properties of PLA and the PVA/MWCNTs composite materials.

Samples	MFR(g/10 min)
PLA	18.8 ± 2.3
PVA/MWCNT-1%	20.0 ± 2.8
PVA/MWCNT-3%	24.4 ± 3.1
PVA/MWCNT-5%	27.5 ± 3.9
PVA/MWCNT-7%	8.6 ± 1.2
